# Cannabinoid interactions with ion channels and receptors

**DOI:** 10.1080/19336950.2019.1615824

**Published:** 2019-05-15

**Authors:** Abeline Rose Watkins

**Affiliations:** Department of Biomedical Physiology and Kinesiology, Simon Fraser University, Burnaby, BC, Canada

**Keywords:** Cannabidiol, CBD, ion channels, review, membrane fluidity, cannabinoids

## Abstract

Cannabidiol (CBD), the non-psychoactive component of *Cannabis sativa*, acts on a diverse selection of membrane proteins with promising therapeutic potential in epilepsy and chronic pain. One such protein is the voltage-gated sodium channel (Na_v_). CBD shows a lack of specificity for sodium channels; however, the method of interaction is still unknown. In this review, we will outline the studies that report reproducible results of CBD and other cannabinoids changing membrane channel function, with particular interest on Na_v_. Na_v_ are implicated in fatal forms of epilepsy and are also associated with chronic pain. This makes Na_v_ potential targets for CBD interaction since it has been reported to reduce pain and seizures. One potential method of interaction that is of interest in this review is whether CBD affects channel function by altering lipid bilayer properties, independent of any possible direct interaction with membrane channels. CBD’s ability to interact with its targets is a novel and important discovery. This discovery will not only prompt further research towards CBD’s characterization, but also promotes the application of cannabinoids as potentially therapeutic compounds for diseases like epilepsy and pain.

## Introduction

**Figure 1.**
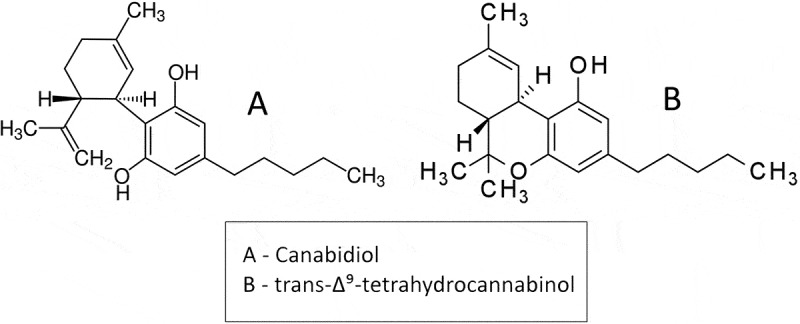
Line drawing chemical structures of cannabidiol (CBD) and trans-Δ⁹-tetrahydrocannabinol (THC)

*Cannabis sativa*, a plant known more commonly as marijuana, has become widely popular due to its induced psychological and euphoric states in an individual who ingests or smokes the plant. More interestingly, the cannabinoids that cause these desired effects also have potential health applications which have been seen to improve a variety of symptoms such as neuropathic pain, seizures, social defects, brain damage from stroke, and lung function in inflammatory lung disease [1–8]. The most popularly used compounds recreationally and therapeutically, Cannabidiol (CBD) and trans-Δ⁹-tetrahydrocannabinol (THC)(Figure 1), are classified as phytocannabinoids since they naturally occur from the Cannabis plant [9]. Living organisms also biologically synthesize their own cannabinoids, called endocannabinoids, which interact with the organism’s endocannabinoid system (ECS) [9]. The ECS regulates hormones associated with reproductive functions and stress, and are localized in the brain, endocrine system, and immune tissues [10]. To target these functions, non-natural structural analogs of endo- and phytocannabinoids, known as synthetic cannabinoids, are produced in laboratories to interact with and regulate the ECS [9].

The ECS includes two endogenous cannabinoid receptors, CB1 and CB2. The interactions between cannabinoids and cannabinoid receptors have been extensively studied [11,12], but here, we focus on different interactions. Although prevalent thought held that cannabinoids bind exclusively to CB1 and CB2, this has been proven to be false by countless studies showing interactions between cannabinoids and other membrane proteins [13–18]. Whereas some cannabinoids still interact with CB1 and CB2, they also interact with a large range of targets including other receptors, transporters, enzymes, cellular structures, membranes, and ion channels [19,20]. The intent of this review is to focus on the last two listed targets; cellular membranes and ion channels. To break it down further, the cannabinoid of interest is CBD due to the lack of data surrounding its effects and the popular and widespread use of the compound in modern day society. CBD is also different from other cannabinoids due to some unique effects it exhibits, such as is its inverse antagonism of CB2 when compared to THC [21]. In this review, we outline the known interactions of CBD and other cannabinoids on molecular targets, particularly voltage-gated sodium channels (Na_v_), and discuss potential mechanisms of CBD besides the known interactions with CB1 and CB2.

## Discussion

### Sodium channel interactions

Na_v_ are ion conducting transmembrane proteins that allow the passage of sodium ions (Na+) along their electrochemical gradient [22]. Once channels are activated by a depolarizing membrane potential, sodium flows into the intracellular environment due to a lower concentration of Na+ on the inside of the cell vs. outside [22]. Within milliseconds of channel opening, an inactivation gate closes and the channel becomes impermeant, preventing further flow of ions through the central pore [22].

A common theme in cannabinoid research on Na_v_ is the ability of the drug to induce channel block, or in other words, inhibit Na+ current [14,15]. 2-AG ether, an endocannabinoid, was found to decrease peak Na+ current in frog parathyroid cells which do not express CB1 and CB2. These data suggest that cannabinoids can interact with more than just their respective receptors to produce current changes in ion channels. Basal properties of the frog parathyroid cells were unaffected when 50 μM of 2-AG ether was applied to the extracellular environment of the cells; however, a 36 ± 8% decrease of peak current at −24mV was reported to be irreversible due to the channels’ inability to recover its initial peak current after a saline solution washout [15]. WIN 55,212–2, an aminoalkylindole derivative that acts on target proteins in a similar way to THC and CBD, was also tested on Na_v_ and produced left shifts in the V1/2 of activation by 11.7 ± 1.6 mV and the V1/2 of inactivation by 17.5 ± 1.9 mV [15]. This shift in activation/inactivation resulted in the channel becoming inactive at more negative potentials, rendering fewer channels available to activate at the −24mV potential recorded for peak current when exposed to the drug vs its native peak current alone. Another study on Na_v_ found introducing various synthetic cannabinoids and endocannabinoids to the extracellular environment to also inhibit current, but they did not test CBD [14]. Both studies demonstrated that cannabinoids affect Na_v_ function with changes in biophysical properties of activation/inactivation in addition to the current block. With some insight on how cannabinoids affect sodium channels, research can move a step closer in determining other cannabinoid interactions throughout the body.

Based on previous studies reporting Na_v_ block with drugs similar to CBD, a study of CBD effects on Na_v_ was done to determine whether CBD directly or indirectly affects sodium channels [20]. Interestingly, CBD had no specificity to any of Na_v_1.1, 1.2, 1.3, 1.4, 1.5, 1.6, 1.7, or mNa_v_1.6 and also produced similar inhibitory effects with ~90% inhibition of sodium conduction in Na_v_1.3 when 3.3 µM CBD was applied [20]. When looking for a mechanism of interaction, potency of CBD was tested in a F1763A-mutant Na_v_ channel (altering the local anesthetic receptor site) to find only a slight decrease in potency suggesting that CBD does not inhibit at the classic pore-binding sites for Na_v_1.1 [20]. This suggests that CBD may interact through a mechanism that is common among an array of different channels such as fenestrations, or by changes to the membrane environment in which channels are situated.

### Other channel interactions

In support of cannabinoids being non-specific for their targets, these compounds and their synthetic derivatives were reported to interact with other membrane channels. One study produced a concentration-dependent decrease in peak current of K_v_1.2 in B82 transfected fibroblasts with complete inhibition of current when 750nM of Anandamide (an endocannabinoid) or 1.5 µM of Δ9-THC (a phytocannabinoid) were introduced [16]. That study eliminated CB1 as the method of interaction since both cannabinoids displayed no change in potency in the presence of SR141716A (a common CB1 inhibitor) [16]. G-protein-coupled receptors were also not likely to be involved since recordings performed with nucleotide-free intracellular solutions had no effect on current changes [16].

The effects of cannabinoids on ligand-gated potassium channels were also studied. Anandamide decreased current of ATP gated potassium channels (K_ATP_) during cromakalim experiments (cromakalim induces outward current in follicle-enclosed oocytes with K_ATP_) with 8.1 µM anandamide preventing current induction by 50%, with maximum block (84 ± 7%) achieved with 100µM [23]. CB1 antagonist SR141716A and CB2 antagonist SR144528 (both at 1µM) did not affect anandamide inhibition of cromakalim-activated currents, again suggesting these receptors are not required to modify channel currents [23]. More interestingly, anandamide does not compete with the cromakalim binding site in cromakalim-activated K_ATP_, suggesting the endocannabinoid is not a competitive inhibitor for ligand-gated potassium channels [23]. Competitive inhibition presents with altered EC50 values and unaffected Vmax values in substrate–velocity curves, while cromakalim EC50 values in the presence of CBD were unaffected and Vmax values decreased [23]. An altered Vmax with a constant EC50 classifies a non-competitive inhibitor, such that the inhibitor does not compete with the ligand for the active site of the target but instead uses another site on the protein. Again, these results suggest cannabinoids interact with membrane channels without using CB1 and CB2 and expands cannabinoid effects to an additional class of membrane channels – ligand-gated channels. This addition extends the range of molecular targets available to cannabinoids which suggests even more proteins could be affected and manipulated for therapeutic use.

In addition to potassium channels, Δ9-THC and anandamide also induce concentration-dependent potentiation of currents mediated by α_1_ homomeric and α_1_β_1_ heteromeric glycine receptors (GlyRs) [24]. Tested in both X. laevis oocytes and isolated ventral tegmental area neurons (neurons involved in the reward pathway of the brain) with 3µM of glycine to activate the receptor, THC was more potent than anandamide [24]. THC EC50 values for decreased potential current were 73nM and 115nM for heteromeric GlyRs and native GlyRs, respectively [24]. These concentrations make the results more physiologically relevant since they are within pharmacological ranges that induce psychotropic and anti-nociceptive effects in humans [24]. This suggests large concentrations are not required to cause a significant effect on GlyRs and promotes the application to human use as a therapeutic target to manipulate reward pathway problems such as addiction since the effect was seen in ventral tegmental area neurons. Structurally similar to GlyRs, GABA_A_ receptors were also tested with both anandamide and THC yet no significant effects were observed [24].

More specifically, CBD also affects a host of different membrane proteins besides Na_v_. Similar to THC’s modulation of GlyRs, CBD also activates both α_1_ homomeric and α_1_β_1_ heteromeric glycine receptors; however, the EC50 values for direct activation by CBD are much higher [25]. TRPV1 and other TRP channel subfamilies were activated and desensitized though patch clamp analysis on HEK293 cells involving CBD, while TRPM8 was inhibted [17,26,31]. In addition to Calcium currents being affected through TRP channels, CBD also inhibits T-Type Calcium Channels Ca_v_3.1, Ca_v_3.2, and Ca_v_3.3 in the same model system [27]. Other CBD research reported interactions with voltage-dependent anion-selective channel protein 1 (VDAC1), G-protein coupled receptor protein 55 (GPR55), and adenosine reuptake via Equilibrative Nucleoside Transporter (ENT1) while potentially also activating A_2A_ receptors [18,28,29]. Serotonin receptors H5-HT1A and 5-HT2A were also targets for CBD modulation [30].

The considerable variety of targets affected by cannabinoids leads us to suggest there may be a similar underlying mechanism of action for CBD and other cannabinoids. Table 1 shows that CBD IC50s/EC50s in particular are also considerably similar between all its targets (with the exception of GlyRs). The need for further research in this area is necessary to describe the mechanism of CBD to understand its potential applications in human health.

**Table 1.** Quantitative results of Cannabidiol’s membrane protein interactions by target, cell type, and IC50 or EC50. Missing IC50 values indicate that dose–response curves were not produced, but significant effects were demonstrated.

	Target	Cell type	IC50 (µM)
Channels	Na_v_1.1	HEK-293	2.0 ± 0.1 [20]
	Na_v_1.2	HEK-293	2.9 ± 0.1 [20]
		iPSCs	1.3 ± 0.1 [20]
	Na_v_1.3	HEK-293	3.3 ± 0.1 [20]
	Na_v_1.4	HEK-293	1.9 ± 0.1 [20]
	Na_v_1.5	HEK-293	3.8 ± 0.2 [20]
	Na_v_1.6	HEK-293	3.0 ± 0.1 [20]
	Na_v_1.7	HEK-293	2.9 ± 0.1 [20]
	NaChBac	HEK-293	1.5 ± 0.2 [20]
	K_v_2.1	HEK-293	3.7 ± 0.8 [20]
	TRPM8	HEK-293	0.06 ± 0.01 [26]
	Ca_v_3.1	HEK-293	0.813* [27]
	Ca_v_3.2	HEK-293	0.776* [27]
	Ca_v_3.3	HEK-293	3.63* [27]
	VDAC1	Planar lipid bilayer	–[18]
Transporters	Adenosine uptake via ENT1	EOC-20 microglia	0.12 [28]
	Thymidine uptake via ENT1	EOC-20 microglia	0.19 [28]
Receptors	GPR55	HEK-293	0.445 ± 0.067 [29]
	H5-HT1aR	CHO Cells	–[30]
	5-HT2aR	CHO Cells	–[30]
	Target	Cell Type	EC50 (µM)
Channels	TRPV1	HEK-293	1.0 ± 0.1[26]
	TRPV2	HEK-293	1.25 ± 0.23[26]
	TRPA1	HEK-293	0.11 ± 0.05[26]
Receptors	α_1_ homomers GlyRs	n/a	132.4 ± 12.3[25]
	α_1_β_1_ heteromers GlyRs	n/a	144.3 ± 22.7 [25]

^*^ Numbers calculated from pEC50 values

### Membrane interactions

As noted above, cannabinoids interact with a diverse group of targets including voltage-gated channels, ligand-gated channels, and GPCRs, some even without specificity. How do these compounds cause these effects in such a wide range of targets? A common feature among all these channels is that they directly associate with the lipid membrane in which they are expressed. Changes in membrane fluidity alter conformational changes between conducting (open) and nonconducting (closed) states of membrane channels [32], so changes to the membrane itself have an “indirect” effect on channel function. One possible hypothesis regarding cannabinoid interactions is that it could alter the properties of the lipid bilayer.

When a channel sits in the bilayer, the two lipid monolayers facilitate the movement of the channel between different conducting states through changes in monolayer compression and bending [33]. The membrane’s ability to compress and change shape is directly related to the rigidity of the membrane which, in turn, controls channel conformation and gating kinetics in different membrane compositions [33]. Gramicidin channels can be used to test membrane fluidity [34–36]. An increase in fluidity permits phospholipid monolayers to more easily bend and increase the rate at which two gramicidin half channels combine to form a leak producing pore [33]. Measuring the resultant changes in membrane resistance can be extrapolated to assess changes in membrane fluidity. Increasing bilayer rigidity by incorporating higher concentrations of cholesterol into the bilayer was found to shift channel activation towards more positive potentials [33]. Using non-physiological amphiphiles to increase fluidity in the membrane, β-octyl-glucoside (βOG) at 5 mM and reduced Triton X-100 (TX100) at 30 μM, reversibly inhibited Na_v_ currents (decrease peak current) [37]. βOG and TX100 were suggested to promote the steady-state inactivation of Na_v_ since 2.5 mM βOG or 10 μM TX100 changed the initial voltage of activation by −8.3 ± 1.6 mV or −9.8 ± 1.0 mV, respectively, and the initial slope factor of activation by +2.2 ± 0.6 mV or +1.7 ± 0.3 mV, respectively [37]. These results promote the idea that CBD could interact with the membrane in a way to increase fluidity as its method of protein interaction. As previously noted, CBD inhibits current, similar to βOG and TX100, and shifts channel activation towards negative potentials, opposite to the effect seen upon adding cholesterol to the membrane. This suggests that CBD increases membrane fluidity as its interaction mechanism.

Since CBD and other derivatives have been seen to affect various membrane channels, and membrane channels are affected by changes in membrane fluidity, this raises the hypothesis that CBD interacts with many targets by disrupting the membrane they are embedded in. With limited knowledge surrounding this idea, expanding CBD research to include how this compound can alter membrane properties would open up our understanding of this compound and possibly promote its use as a potentially therapeutic compound.

## Conclusion

Whereas cannabinoids initially were thought to interact with their specific CB1 or CB2 receptors, a growing body of research demonstrates that these chemical compounds can interact with a wide range of other targets such as sodium, potassium, and calcium channels, and serotonin and glycine receptors. Cannabinoids interact with these targets independently of CB1 or CB2 receptors and without specificity. Other studies have reported that CBD does not require G-proteins to activate changes in channel function, while some studies concluded that cannabinoids do not need to compete for binding sites with other ligands to make an impact. With these results in mind, and while also considering the non-polar nature of cannabinoids, one mechanism of interaction may be that they insert into the lipid bilayer and impact membrane fluidity. In conclusion, since changes in membrane properties affect the function of transmembrane channels, cannabinoids may change channel function by their effect on lipid bilayer properties instead of (or in addition to) directly interacting with the channels. Although this review sought to compile research on CBD interaction and suggest a potential mechanism of interest, more research is needed to determine the exact mechanism of how CBD and other cannabinoids interact with their various molecular targets. If anything, this review seeks to validate further cannabinoid research.
